# Comparison of Analgesic Efficacy between Epidural and Perineural Administration of Autologous Conditioned Serum in the Conservative Treatment of Low Back Pain Due to Lumbar Degenerative Disc Disease: A Randomized, Open-Label, Controlled Clinical Trial

**DOI:** 10.3390/brainsci13050749

**Published:** 2023-04-30

**Authors:** Piotr Godek, Beata Szczepanowska-Wolowiec, Dominik Golicki

**Affiliations:** 1 Sutherland Medical Center, 04-036 Warsaw, Poland; 2 Institute of Health Sciences, Collegium Medicum, The Jan Kochanowski University, 25-317 Kielce, Poland; beatawolowiec@op.pl; 3 Department of Experimental and Clinical Pharmacology, Medical University of Warsaw, 02-091 Warsaw, Poland; dominik.golicki@wum.edu.pl

**Keywords:** Orthokine, interleukin-1 inhibitor, autologous conditioned serum, low back pain, degenerative disc disease, lumbar spine

## Abstract

Lumbar degenerative disc disease (LDDD) is widely acknowledged as a significant contributor to low back pain (LBP), which is a prevalent and debilitating health condition affecting millions of individuals worldwide. The pathogenesis of LDDD and associated pain mechanisms are thought to be mediated by inflammatory mediators. Autologous conditioned serum (ACS, Orthokine) may be used for symptomatic treatment of LBP due to LDDD. This study aimed to compare the analgesic efficacy and safety of two routes of ACS administration, perineural (periarticular) and epidural (interlaminar), in the conservative treatment of LBP. This study used an open-label, randomized, controlled trial protocol. A group of 100 patients were enrolled in the study and randomly allocated into two comparative groups. Group A (*n* = 50) received the epidural (interlaminar) approach—2 ultrasound-guided injections as control intervention (each containing two doses of ACS—8 mL). Group B (*n* = 50) received the perineural (periarticular) approach—2 ultrasound-guided injections as experimental intervention at 7-day intervals (the same volume of ACS). Assessments consisted of an initial assessment (IA) and control assessments at 4 (T1), 12 (T2), and 24 (T3) weeks after the last intervention. Primary outcomes comprised Numeric Rating Scale (NRS), Oswestry Disability Index (ODI), Roland Morris Questionnaire (RMQ), and Euro Quality of Life—5 Dimensions–5 Levels (EQ-5D-5L): Index, Visual Analogue Scale (VAS), and Level Sum Score (LSS). Secondary outcomes included differences between groups in specific endpoints for the above-mentioned questionnaires. In conclusion, this study revealed that both perineural (periarticular) and epidural ACS injections tended to perform in a very similar way. Both routes of Orthokine application show significant improvement in the primary clinical parameters, such as pain and disability, and therefore, both methods can be considered equally effective in managing LBP due to LDDD.

## 1. Introduction

Lumbar degenerative disc disease (LDDD) is a common cause of low back pain (LBP), which is a significant and prevalent health issue experienced by many individuals [1,2]. LBP can be caused by various conditions, including lumbar disc herniation (LDH) and intervertebral disc degeneration (IDD), which can lead to lumbar spinal stenosis (LSS). However, these conditions have different underlying causes [3].

Lumbar disc herniation (LDH) caused by herniated nucleus pulposus (NPH) occurs when the soft center of a spinal disc pushes through a crack in the outer layer of the anulus fibrosus and irritates nearby nerves [4]. The pain associated with HNP is often described as sharp and shooting, and it can radiate down the leg. In addition to pain, symptoms of HNP can include numbness, tingling, and muscle weakness [5]. In turn, intervertebral disc degeneration (IDD) occurs when the discs between the vertebrae of the spine start to break down and lose their ability to absorb compressive forces. This can lead to inflammation, pain, and stiffness in the back [6]. The pain associated with IDD is typically described as a dull ache, and it may be worse in the morning or after sitting for extended periods [7].

To sum up, LDDD can manifest with a variety of symptoms, including chronic low back pain, radicular pain, and sensory disturbances, such as numbness or tingling sensations in the legs [8,9]. LDDD is commonly diagnosed using imaging techniques such as CT or MRI, which can reveal a loss of disc height, disc herniation, or other changes in the structure of the intervertebral discs [10]. It is estimated that in the global population, the 1-year incidence of the first-ever episode of LBP ranges between 6.3% and 15.4%, while the estimated 1-year incidence of any episode of LBP ranges between 1.5% and 36% [11].

Although most cases can be treated conservatively, the golden standard of LBP due to LDDD management is still a matter of debate [12], despite the effectiveness of conservative treatments such as physical therapy, exercise, and pain medications in managing LBP due to LDDD in most cases [13]. Although some clinicians may recommend surgical intervention, others may prefer more conservative approaches, and the choice of management approach may depend on various factors, such as the severity of symptoms, patient preferences, and available resources [14,15]. Thus, the establishment of a golden standard for LBP due to LDDD management is still under debate.

Inflammatory mediators are considered to contribute to the pathogenesis of pain associated with LDDD degeneration and associated pain mechanisms [16]. Therefore, assuming that inflammatory reaction and significant edema are present around the affected spinal segment, one of the most popular options in the armament of pain caused by LDDD conservative management is epidural steroid injections (ESI). Numerous studies are confirming the temporary reduction in pain through ESI, but in addition to well-known side effects, there is a risk of microembolism when using crystalline steroids; thus, ultimately, noncrystalline steroids in epidural space are recommended [17,18,19].

A potential alternative for ESI may be an autologous conditioned serum (ACS, Orthokine), introduced in 2003 by Peter Wehling and Julio Reinecke. The concept is based on the anti-inflammatory action of several cytokines and proteins through a competitive blocking of interleukin-1 receptors (interleukin 1- Receptor agonist, IL-1Ra). A straightforward technological method of IL-1Ra release was developed by stimulating blood monocytes to secrete in specially developed tubes containing glass beads. Further factors secreted are anti-inflammatory cytokines IL-4, IL-10, and numerous growth factors, such as hepatocyte growth factor (HGF), insulin-like growth factor-i (IGF-1), transforming growth factor beta (TGF-β), platelet-derived growth factor (PDGF), fibroblast growth factor (FGF), and epidermal growth factor (EGF). These agents act synergistically with IL-1Ra and positively affect the regeneration and healing processes. In addition, the production of pro-regenerative factors during blood incubation is well-documented [20,21,22,23].

It has recently become clear that pain resolution is an active process that requires transcriptional, metabolic, and cellular processes [24]. Transient upregulation of inflammation mechanisms improves the prospect of chronic pain resolution. Hence, the involvement of inflammatory and proresolving mediators is a delicately honed and timed process. Against this background, it is of concern that short-acting nonsteroidal anti-inflammatory drugs (NSAIDs), corticosteroids, and local anesthetics have been described as potentially cytotoxic and/or are also agents leading to chronified changes. Furthermore, their mode of action appears to fall short of a regenerative effect. ACS lacks these unwanted effects. It has shown therapeutic potency in numerous pathologies (e.g., musculoskeletal, neurological, and skin), demonstrated stimulation of mesenchymal stem cells [25], and accelerated the healing of injuries. A particular feature was revealed by Shirokova et al. [26], who found exciting signs that ACS may remedy mitochondrial dysfunction, shown by a reduced reactive oxygen species (ROS)-footprint load in the knee joint.

The features of metabolic stress and chronic mitochondrial dysfunction can cause ROS overload. This is relevant for degenerating the intervertebral disc, nervous tissue, and other tissues. It is, therefore, of interest that ACS delivers elevated levels of humanin, one of the multiple mitochondrial-derived peptides (MDP) discovered in the last decade. Evidence shows that MDP may modulate adaptative responses to metabolic stress and crucial energy supply for tissue healing [27]. Measurements of further MDPs in ACS are on the way.

In the present study, the authors aimed to clarify if ACS is more effective when given in the vicinity of pain generators (inside the spinal canal, epidural) vs. perineural (outside of the spinal canal, upon the articular column, and in the surroundings of the nerve roots). The risk–benefit ratio could shift to a new paradigm of ACS administration. The experience with perineural ACS has shown promising results; however, the main question remains whether we can achieve further improvement, particularly in cases of disc-related inflammation.

This study aimed to verify the value of the pain-resolving effect of ACS in LDDD and to evaluate the efficacy of experimental perineural (periarticular) administration of Orthokine compared with the epidural (interlaminar) approach. It was taken into account that the disc-related inflammation in the spinal canal affects important pain generators located there (meningeal branches of the spinal nerves and dorsal root ganglia).

## 2. Materials and Methods

### 2.1. Study Design

This therapeutic, investigator-initiated trial was designed as a prospective, two-armed, controlled, randomized, open-label, single-center, interventional clinical study. The experimental group underwent an intervention of ultrasound-guided perineural injection of Orthokine. The control group received an intervention of ultrasound-guided epidural injection of Orthokine. This study follows the CONSORT (Consolidated Standards of Reporting Trials) guidelines.

### 2.2. Ethical Consideration

The study received ethical approval from the Bioethics Committee at the Faculty of Health Sciences of Jan Kochanowski University in Kielce (approval no. 4/2021, approval date: 12 January 2021). The trial was registered on ClinicalTrials.gov (NCT04734327) on 20 January 2021 (Initial Release) and was last updated on 16 March 2021. The study was conducted in compliance with Good Clinical Practice guidelines and the Declaration of Helsinki. The protocol ID for this study is SMC2021001.

### 2.3. Participants

The study was performed monocentrically in Sutherland Medical Center (SMC), Warsaw, Poland. All eligible male and female patients aged at least 18 years with LBP due to LDDD who presented themselves at the study center were offered participation in the study. Only patients with LDDD in the course of intervertebral disc degeneration in the lumbar spine confirmed by MRI were included with accompanying symptoms such chronic LBP and radicular pain with pain radiation to the buttocks, legs, or feet, as well as stiffness and numbness or tingling in the legs. Further inclusion criteria for participation in the study were the absence of contraindications to ACS injections (such as hemorrhagic diathesis, use of anticoagulants, or skin lesions) and provision of written informed consent.

Exclusion criteria for study enrollment were the presence of severe neurological deficits requiring surgery and discopathy with other origins, such as trauma, spondylolisthesis, cancer, infection, or systemic inflammatory diseases. Additional exclusion criteria included previous surgical treatment in the lumbar spine and a mental state that prevented cooperation during injections (such as a phobia of injections).

### 2.4. Interventions

Blood samples of 4 × 10 mL were collected from each of the patients enrolled in the study. A CE-labelled medical device (Orthogen Lab Services GmbH, Düsseldorf, Germany) was used to collect 4 × 10 mL of whole blood to process cell-free ACS. Patient details were appropriately labeled on the device. The blood-filled device was then left in a controlled incubator for a prolonged coagulation period (9 h at 37 °C) and then centrifuged at 3000× *g* for 10 min. The extracted serum (4 mL per device) is cell-free autologous conditioned serum, known as ACS. It contains growth factors, cytokines, and further pro-regenerative factors released during extended coagulation.

Two treatments of a double dose of 8 mL ACS each were given 7 days apart by the same operator. The first treatment was administered immediately after serum preparation. Until the second treatment, ACS was stored frozen at −18 °C or colder.

It should be noted that for patients with multilevel discopathy, the autologous conditioned serum (ACS) was administered to the level of discopathy that was most predominant, meaning the level where the herniation was the greatest or the most prominent lumbar spinal stenosis was correlated with clinical signs. When administered epidurally, the serum was distributed throughout the spinal canal of the lumbosacral region, so the specific level of injection did not make a significant difference.

It should also be explained that the decision to use the ultrasound-guided injection technique instead of the C-arm method for the epidural injections was based on the recent experience of our clinic, which has been demonstrated in our previously published articles. We have extensive expertise in performing ultrasound-guided injections and found it to be a safe and effective method for delivering epidural injections for LBP. We also found that the ultrasound-guided technique provides excellent visualization of the relevant anatomical structures and accurate placement of the injection, without exposing the patient or staff to the additional radiation associated with the use of the C-arm.

**Figure 1:**
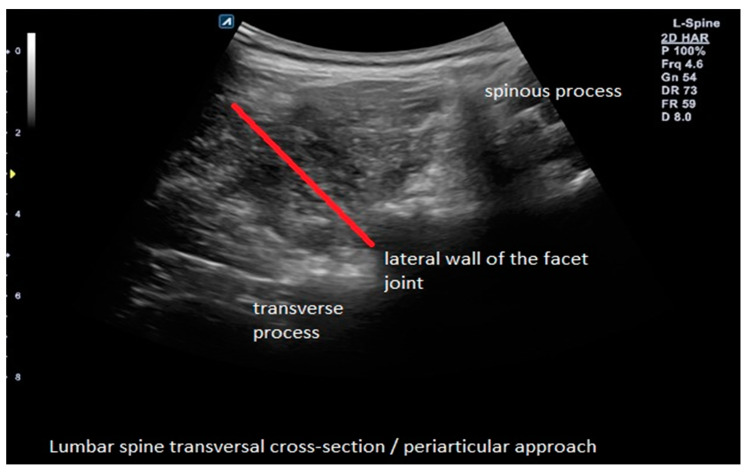
Example of ultrasound-guided perineural (periarticular) ACS injection with needle trajectory in the experimental group (B).

The experimental group (B) received an ultrasound-guided perineural (periarticular) injection where the needle is guided upon the lateral wall of the articular process (as it is performed in the medial branch block) so that the injectate is spread indirectly over the nerve root, which it is not the same as a transforaminal approach because ACS is localized around facet joints of the affected segment (Figure 1).

**Figure 2:**
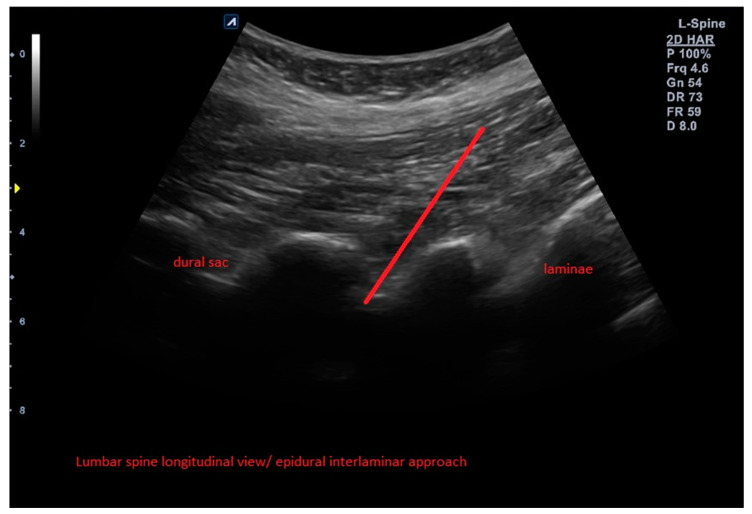
Example of ultrasound-guided epidural ACS injection with needle trajectory in the control group (A).

The control group (A) as an active comparator received an ultrasound-guided epidural (intralaminar) injection of Orthokine into the epidural space (interlaminar approach) above the affected segment (Figure 2).

### 2.5. Outcomes

All outcomes were measured at baseline (IA) as well as after weeks 4 (T2), 12 (T3), and 24 (T3). The following evaluation parameters were selected for the measurement of efficacy: Numeric Rating Scale (NRS: 0–10), Oswestry Disability Index (ODI: 0–50), and Roland Morris Questionnaire (RMQ: 0–24). The Euro Quality of Life—5 Dimensions–5 Levels (EQ-5D-5L) and its group variants were used to measure quality of life: Index (−0.590–1.00), Visual Analogue Scale (VAS: 0–100), based Level Sum Score (LSS: 5–25), mobility, self-care, usual activities, pain/discomfort, and anxiety/depression.

Three primary outcome criteria for each group were defined: (1) change in EQ-5D-5L Index from baseline to 24 weeks, (2) change in ODI from baseline to 24 weeks, and (3) change in RMQ score from baseline to 24 weeks.

Secondary evaluation criteria are all other measurements at each time point. After the study started, no important changes to methods were introduced. Safety was assessed based on the adverse event form.

### 2.6. Sample Size

The sample size was determined by clinic recruitment capacity rather than calculation. The enrolled sample size of 100 (2 × 50) patients makes it possible to detect a statistically significant effect size of at least 0.56 in paired two-tailed t-tests with an alpha error of 0.05 and a power of 80%. This sample size is also sufficient to demonstrate the noninferiority of the perineural group compared with the epidural group with a power of 80%, a lower bound of the one-sided 97.5% confidence interval, and a margin of noninferiority of −1.

### 2.7. Randomization and Blinding

Randomization using a random number generator achieved structural equality between the two arms. The allocation was performed using permutated blocks of variable lengths in a 1:1 ratio. The study is open-label (no blinding) for the observer and injector.

### 2.8. Statistical Methods

Statistical analyses were conducted using descriptive techniques (i.e., frequency tables, means, standard deviations, and effect sizes) and inferential analyses using appropriate significance tests and confidence intervals. Missing values were not imputed. Probabilities for continuous data were calculated using a two-tailed t-test for paired and unpaired data, respectively. Probabilities for confidence tables were calculated using a chi-squared test. All tests were performed with a global significance level of α = 0.05 and a null hypothesis that the mean effect is 0. For the noninferiority test, a noninferiority margin of 25% was defined for all variables and compared with the lower 2.5% confidence interval of the perineural group. The primary and secondary endpoints were analyzed according to the intention-to-treat (ITT) principle. Results were interpreted on a confirmatory basis. Safety was assessed exploratively. Statistical analysis was performed using SPSS version 24.0.

## 3. Results

### 3.1. Participants

**Figure 3:**
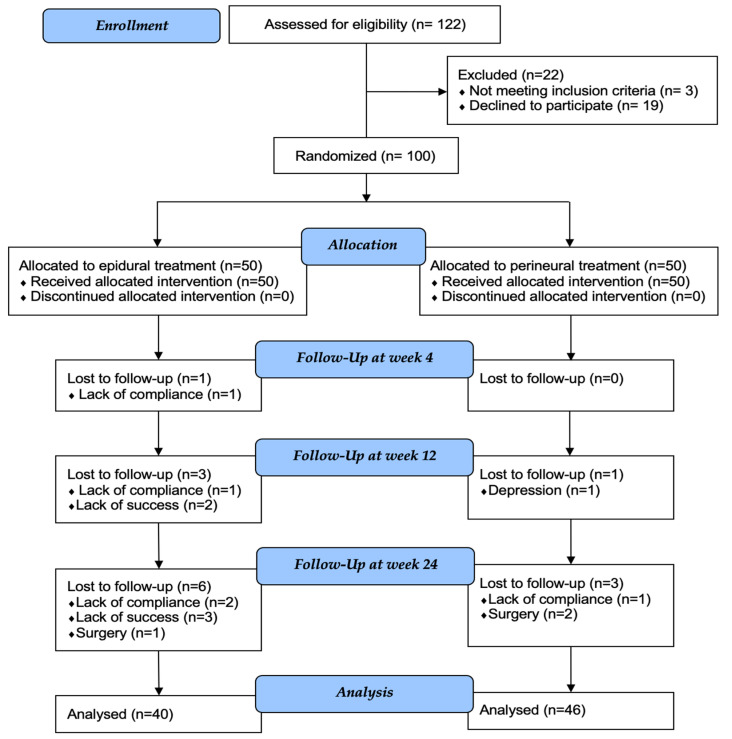
CONSORT flow diagram showing enrollment, allocation, follow-up, and participants included in statistical analysis.

From February to May 2021, 122 patients were assessed for eligibility. Of these, 3 patients did not meet the inclusion criteria, and 19 refused to participate in the study. As a result, 100 patients were assigned to treatment and enrolled in the study. All of these patients completed the entire course of treatment (Figure 3).

### 3.2. Baseline Data

Baseline data for demographics, confounders, and pre-existing conditions showed a balanced randomization outcome. On average, patients in the epidural group of Orthokine administration were 1.5 years older. The proportion of men was slightly higher. The proportion of hernia prolapse type was moderately higher in the perineural group (Table 1).

Baseline data for all evaluation parameters and all time points (baseline and weeks 4, 12, and 24) are shown in Table 2.

### 3.3. Primary Outcome (Change in EQ-5D-5L Index from Baseline to 24 Weeks)

**Figure 4:**
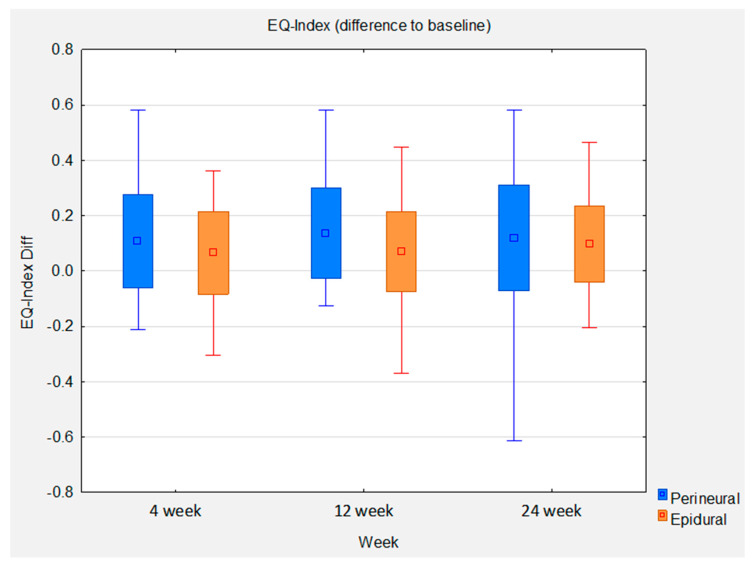
Box whisker plots of EQ-Index difference to baseline between groups at control time points.

Statistical analysis of the EQ-5D-5L Index between baseline and 24 weeks shows a mean improvement of 0.0969 in the epidural group. This gives an effect size of 0.7 (*p* = 0.0001). The mean improvement in the perineural group is higher, at 0.119. However, the effect size is lower, at 0.62 (*p* = 0.0001), due to the slightly higher standard deviation in this group. The between-group comparison shows a slight mean difference of 0.022 favoring the perineural group. The between-group effect size is 0.13. The statistically significant noninferiority of the perineural group compared with the epidural group at week 12 could not be reproduced at week 24, as the mean improvement at week 12 of 0.067 favored the perineural group, resulting in an effect size of 0.44 (*p* = 0.0359) (Figure 4).

### 3.4. Primary Outcome (Change in Oswestry Disability Index from Baseline to 24 Weeks)

**Figure 5:**
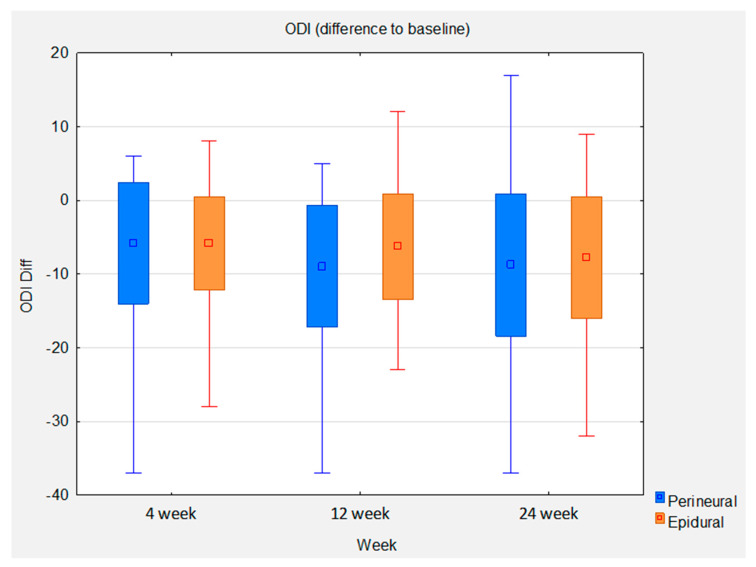
Box whisker plots of ODI difference to baseline between groups at control time points.

Statistical analysis of the ODI between baseline and 24 weeks shows a mean improvement of 7.8 in the epidural group. This gives an effect size of 0.95 (*p* < 0.0001). The mean improvement in the perineural group is higher at 8.78. However, the effect size is slightly lower at 0.91 (*p* < 0.0001) due to the slightly higher standard deviation in this group. The between-group comparison shows a slight mean difference of 0.98 in favor of the perineural group. The between-group effect size is 0.11. The statistically significant noninferiority of the perineural group compared with the epidural group at week 12 was replicated at week 24 (Figure 5).

### 3.5. Primary Outcome (Change in Roland Morris Questionnaire Score from Baseline to 24 Weeks)

**Figure 6:**
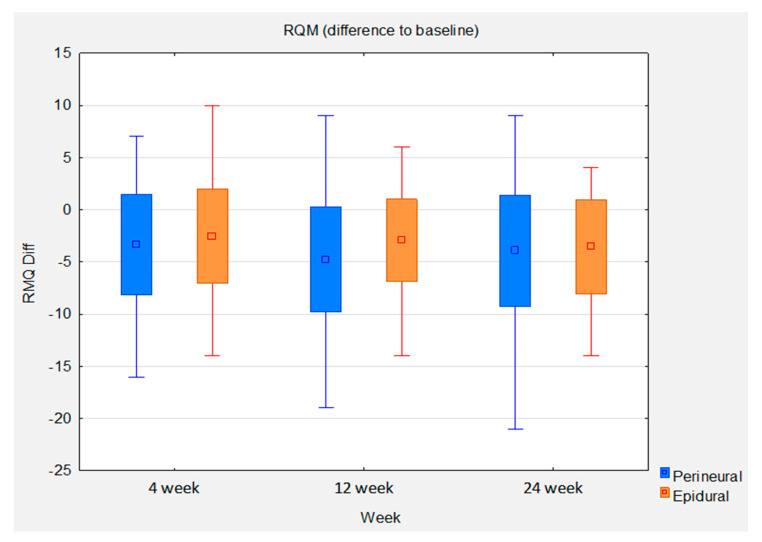
Box whisker plots of RQM difference to baseline between groups at control time points.

Statistical analysis of the RMQ between baseline and 24 weeks shows a mean improvement of 3.55 in the epidural group. This gives an effect size of 0.79 (*p* < 0.0001). The mean improvement in the perineural group is higher at 3.94. However, the effect size is lower at 0.74 (*p* < 0.0001) due to the higher standard deviation in this group. The between-group comparison shows a slight mean difference of 0.38 in favor of the perineural group. The between-group effect size is 0.08. The statistically significant noninferiority of the perineural group compared with the epidural group at weeks 4 and 12 could not be reproduced at week 24 (Figure 6).

### 3.6. Outcomes and Estimations (Intra-Group)

Table 2 shows that the epidural group of Orthokine administration had similar levels of all outcomes at baseline compared to the perineural group of Orthokine administration. It also shows all the follow-up measurements for weeks 4, 12, and 24.

In the within-group comparison, there is a statistically significant improvement in almost all outcome criteria in both arms, with a notable long-term trend toward improvement (Table 3).

### 3.7. Outcomes and Estimations (Superiority between Groups)

Statistical analyses between the epidural and perineural groups consistently show very small effect sizes regarding the superiority of either group (Table 4). This suggests that the two groups tend to perform in a very similar way. A positive Cohen’s d indicates superiority for epidural administration on pain NRS, ODI, RMQ, EQ-5D-5L (mobility, self-care, usual activities, pain/discomfort, anxiety/depression, and level sum score). The reverse is true for EQ-5D-5L VAS and Index. The effect size of the perineural group was superior or almost equal to the epidural group for all outcomes and time points. There was a statistically significant superiority in favor of the perineural group for the EQ-5D-5L mobility (*p* = 0.0432) and Index (*p* = 0.0359) at week 12 and the EQ-5D-5L VAS at weeks 4 (*p* = 0.0364) and 12 (*p* = 0.037). There was no statistically significant superiority in favor of the epidural group for any of the outcome measures.

### 3.8. Outcomes and Estimations (Non-Inferiority)

For the noninferiority test, a noninferiority margin of 25% was calculated for all variables and compared with the corresponding 2.5% confidence interval for the perineural group.

Calculations show the noninferiority of perineural versus epidural for pain NRS (week 24), ODI (weeks 12 and 24), RMQ (weeks 12 and 24), EQ-5D-5L mobility (weeks 12 and 24), EQ-5D-5L VAS (weeks 4, 12, and 24), and EQ-5D-5L (pain/discomfort, anxiety/depression, level sum score, and Index), each at week 12.

### 3.9. Ancillary Analyses

In the subgroup analysis, the 3 primary outcome measures were stratified according to the type of hernia at week 24 to show the within-group effects (Table 5).

### 3.10. Adverse Events

Adverse events were assessed and documented for the entire duration of the study. In addition, the self-report records of the participants were reviewed. Adverse events were assessed by the clinical investigator at each study visit and were summarized according to the intensity and causality of the event. Patients were allowed to withdraw from the study at any time without having to give a reason for doing so.

There were no serious adverse events (SAEs) reported in any of the treatment groups during the 24-week study period. No serious complications were noted during the follow-up. Two cases of transient benign headache in the A group and one case of dizziness in group B were reported shortly after the injection. No infections or neurologic deficits were reported. Both treatment approaches were well-tolerated (Table 6).

## 4. Discussion

Our study provides a foundation for determining the appropriate sample size of research groups based on the effect size that we established. The good tolerability of ACS demonstrated in our study confirms earlier studies and encourages further studies on the efficacy of injection treatment with ACS in larger groups of patients using a placebo, double-blind trial with longer follow-up periods.

Although the conservative treatment of LBP due to LDDD offers a variety of methods, most systematic reviews conclude that there is insufficient evidence to recommend any specific type of nonsurgical treatment [28].

The orthobiologic option is rapidly expanding with platelet-rich plasma (PRP), stem cell therapy (SCT), and ACS (Orthokine). The first encouraging clinical results of Orthokine therapy in the field of spine disorders were published by Becker and al. (2007), showing the superiority of ACS over Triamcinolone epidural perineural injections when comparing three groups of patients with LDDD during 22 weeks of follow-up (ACS, N = 32; Triamcinolone 5 mg, N = 27; and Triamcinolone 10 mg, N = 25) [29].

Other authors obtained promising effects in several small-sample studies without a control group [30,31]. The next stage was introducing ACS into the intervertebral disc to promote its regeneration. This study was conducted with 19 patients by Moser et al. [32], with 3 injections each consecutive week and 6 months of follow-up. The outcomes showed a clinically remarkable and significant reduction in pain (11 out of 19 patients reported at least 50% pain improvement and disability), and the mean improvement was 58% in VAS. No serious side effects and no infections occurred [32].

We decided to introduce interlaminar injections of ACS after analyzing our previously published data about Orthokine treatment in a group of 128 patients with degenerative lumbar spinal stenosis (DLSS) treated by perineural and interlaminar epidural administration. A striking difference in efficacy was rated on the modified McNab scale after 2 and 6 months of follow-up (25% versus 60% and 33% versus 90% of excellent and good outcomes, respectively). The group with the epidural approach was small (*n* = 10) [33]. In the present study, ACS proved its efficacy at every endpoint in reducing pain and disability, with slight differences between the two groups (comparable in terms of demographic data and comorbidities). At 24 weeks, there was a statistically significant difference in all three primary outcome measurements (EQ-5D-5L Index, ODI, and RMQ) in the before/after comparison in both groups (epidural and perineural). Between groups, the perineural group mean was slightly better on all three primary outcome parameters. Statistically significant noninferiority was shown for the ODI in the perineural group. A statistically significant noninferiority was slightly missed for the EQ-5D-5L Index and RMQ variables. Nevertheless, based on this study, regardless of the type and stage of disc injury, the perineural application of ACS is at least not inferior to the epidural for LBP due to LDDD, and considering the risk–benefit ratio, this kind of ACS application should be favored. To the best of our knowledge, there is no report on the epidural interlaminar application of ACS in the treatment of LDDD; so, the strength of our study is to present for the first time an evaluation of the interlaminar epidural route vs. a “conventional” perineural and periarticular approach [34]. At week 24, there was a statistically and clinically significant difference vs. baseline in all three primary outcome scores in both groups (epidural and perineural).

### Study Limitations

The study is limited by the absence of planned sample size calculation for confirmatory hypothesis testing, resulting in an exploratory approach. The study is monocentric, meaning that the findings may not be generalizable to other settings or populations. There is a lack of a comparator drug (placebo or corticosteroid), which limits our ability to compare the effectiveness of ACS treatment to other modalities directly. Subjective questionnaires were used to analyze outcomes, which may be subject to bias or variability in interpretation. A limited follow-up was performed, which may have affected our ability to capture long-term outcomes and potential adverse effects. The dropout rate was 10 out of 50 patients (20%) in the epidural group and 4 out of 50 patients (8%) in the perineural group. However, in the epidural group, 9 out of the 10 patients dropped out of the study due to lack of compliance or lack of success, and only 1 patient dropped out due to a valid reason—surgery. In the perineural group, only one patient dropped out due to lack of compliance, while the other three had valid reasons such as surgery or depression. If noncompliant or failed patients were defined as nonresponders in the response analysis, the results of the study would even more clearly favor the perineural group.

## 5. Conclusions

The present study revealed that both perineural (periarticular) and epidural ACS injections tended to perform in a very similar way. Both routes of Orthokine application showed significant improvement in the primary clinical parameters, such as pain and disability. The therapy revealed a high safety profile. This means that the conventional perineural approach is equal to the epidural interlaminar approach, and considering the potential risk of the epidural interlaminar approach, the perineural route should be favored. Therefore, both methods can be considered equally effective in managing LBP due to LDDD, and the choice of method can be based on factors such as patient preferences and contraindications. Further studies with larger sample sizes and longer follow-up periods are warranted to confirm these findings.

## Figures and Tables

**Table 1:** Demographic characteristics, confounders, and pre-existing conditions.

Character	Epidural Administration		Perineural Administration		*p*-Value *
	N	M ± SD (95% CI of M)	N	M ± SD (95% CI of M)	
Age (in years)	50	47.06 ± 11.86 (43.69 to 50.43)	50	45.52 ± 15.25 (41.19 to 49.85)	0.5742
Height (in cm)	50	173.74 ± 9.36 (171.08 to 176.40)	50	171.46 ± 9.55 (168.8 to 174.17)	0.2308
Weight (in kg)	50	81.10 ± 16.01 (76.55 to 85.65)	50	78.00 ± 14.38 (73.91 to 82.09)	0.3109
Body mass index (BMI)	50	26.85 ± 4.93 (25.45 to 28.25)	50	26.48 ± 4.05 (25.33 to 27.63)	0.6836
Duration of complaints (in weeks)	50	19.10 ± 19.03 (13.69 to 24.51)	50	19.56 ± 19.27 (14.08 to 25.04)	0.9047
	N (%)		N (%)		*p*-Value **
Total	50 (100)		50 (100)		
Gender					0.3196
Female	23 (46)		28 (56)		
Male	27 (54)		22 (44)		
Disease phase					0.4230
Acute (>4 weeks)	7 (14)		10 (20)		
Subacute (4–12 weeks)	21 (42)		15 (30)		
Chronic (>12 weeks)	22 (44)		25 (50)		
Side of discomfort					0.4244
Left side	20 (40)		20 (40)		
Right side	20 (40)		15 (30)		
Both sides	10 (20)		15 (30)		
Type of hernia					0.0951
Bulging	13 (26)		7 (14)		
Extrusion	6 (12)		6 (12)		
Intravertebral	2 (4)		2 (4)		
Prolapse	17 (34)		28 (56)		
Residual	12 (24)		5 (10)		
Sequestration	0 (0)		2 (4)		
Predominant level of hernia					0.5696
Th12/L1	1 (2)		0 (0)		
L2/L3	0 (0)		1 (2)		
L3/L4	2 (4)		1 (2)		
L4/L5	16 (32)		20 (40)		
L5/S1	31 (62)		28 (56)		
Type of discopathy					0.2821
Single level	13 (26)		Single level		13 (26)
Multilevel	37 (74)		Multilevel		37 (74)
Diabetes					0.5597
No	49 (98)		No		49 (98)
Yes	1 (2)		Yes		1 (2)
Peripheral vascular disease					1.0000
No	48 (96)		No		48 (96)
Yes	2 (4)		Yes		2 (4)
Disorders of bone metabolism					0.5597
No	49 (98)		No		49 (98)
Yes	1 (2)		Yes		1 (2)
Polyneuropathy					0.3173
No	50 (100)		No		50 (100)
Yes	0 (0)		Yes		0 (0)

Probability value of unpaired *t*-test * and chi-squared test **. N, total number of cases; M, mean; CI, confidence interval; SD, standard deviation.

**Table 2:** Baseline and follow-up data for all evaluation parameters.

	Epidural		Perineural	
	N	M ± SD (95% CI of M)	N	M ± SD (95% CI of M)
Numeric Rating Scale (NRS)				
Baseline	50	6.10 ± 1.99 (5.53 to 6.67)	50	5.68 ± 2.17 (5.06 to 6.30)
Week 4	49	3.43 ± 2.17 (2.81 to 4.05)	50	3.26 ± 2.18 (2.64 to 3.88)
Week 12	46	3.44 ± 2.52 (2.69 to 4.18)	49	2.62 ± 2.17 (2.01 to 3.24)
Week 24	40	2.80 ± 2.34 (2.05 to 3.55)	46	2.37 ± 2.12 (1.74 to 3.00)
Oswestry Disability Index (ODI)				
Baseline	50	18.26 ± 6.04 (16.54 to 19.98)	50	19.60 ± 8.60 (17.16 to 22.04)
Week 4	49	12.39 ± 6.87 (10.41 to 14.36)	50	13.78 ± 8.90 (11.25 to 16.31)
Week 12	46	11.83 ± 7.26 (9.67 to 13.98)	49	10.31 ± 7.72 (8.09 to 12.52)
Week 24	40	9.65 ± 6.65 (7.52 to 11.78)	46	9.91 ± 10.09 (6.92 to 12.91)
Roland Morris Questionnaire (RMQ)				
Baseline	50	7.40 ± 4.29 (6.18 to 8.62)	50	8.76 ± 5.55 (7.18 to 10.34)
Week 4	49	4.84 ± 4.32 (3.60 to 6.08)	50	5.42 ± 4.63 (4.10 to 6.74)
Week 12	46	4.41 ± 4.29 (3.14 to 5.69)	49	4.06 ± 4.28 (2.83 to 5.29)
Week 24	40	3.08 ± 3.21 (2.05 to 4.10)	46	4.37 ± 5.14 (2.84 to 5.90)
EQ-5D-5L mobility				
Baseline	50	1.94 ± 0.84 (1.70 to 2.18)	50	2.26 ± 0.96 (1.99 to 2.53)
Week 4	49	1.69 ± 0.74 (1.48 to 1.91)	50	1.88 ± 0.85 (1.64 to 2.12)
Week 12	46	1.74 ± 0.77 (1.51 to 1.97)	49	1.63 ± 0.81 (1.40 to 1.87)
Week 24	40	1.58 ± 0.71 (1.35 to 1.80)	45	1.53 ± 0.87 (1.27 to 1.79)
EQ-5D-5L self-care				
Baseline	50	1.74 ± 0.78 (1.52 to 1.96)	50	1.84 ± 1.00 (1.56 to 2.12)
Week 4	49	1.57 ± 0.71 (1.37 to 1.78)	50	1.64 ± 0.83 (1.41 to 1.88)
Week 12	46	1.50 ± 0.75 (1.28 to 1.72)	49	1.57 ± 0.79 (1.34 to 1.80)
Week 24	40	1.38 ± 0.59 (1.19 to 1.56)	45	1.49 ± 0.87 (1.23 to 1.75)
EQ-5D-5L usual activities				
Baseline	50	2.46 ± 0.73 (2.25 to 2.67)	50	2.52 ± 0.91 (2.26 to 2.78)
Week 4	49	2.02 ± 0.75 (1.81 to 2.24)	50	2.06 ± 0.87 (1.81 to 2.31)
Week 12	46	1.94 ± 0.85 (1.68 to 2.19)	49	1.90 ± 0.80 (1.67 to 2.13)
Week 24	40	1.58 ± 0.64 (1.37 to 1.78)	45	1.64 ± 0.83 (1.40 to 1.89)
EQ-5D-5L pain/discomfort				
Baseline	50	3.06 ± 0.74 (2.85 to 3.27)	50	3.08 ± 0.83 (2.84 to 3.32)
Week 4	49	2.45 ± 0.79 (2.22 to 2.68)	50	2.52 ± 0.74 (2.31 to 2.73)
Week 12	46	2.44 ± 0.83 (2.19 to 2.68)	49	2.16 ± 0.77 (1.94 to 2.39)
Week 24	40	2.13 ± 0.76 (1.88 to 2.37)	45	2.18 ± 0.91 (1.90 to 2.45)
EQ-5D-5L anxiety/depression				
Baseline	50	2.02 ± 0.87 (1.77 to 2.27)	50	2.24 ± 1.19 (1.90 to 2.58)
Week 4	49	1.69 ± 0.77 (1.47 to 1.92)	50	1.90 ± 1.05 (1.60 to 2.20)
Week 12	46	1.74 ± 0.95 (1.46 to 2.02)	49	1.59 ± 0.81 (1.36 to 1.83)
Week 24	40	1.58 ± 0.71 (1.35 to 1.80)	45	1.56 ± 0.81 (1.31 to 1.80)
EQ-5D-5L-based Level Sum Score (LSS)				
Baseline	50	11.22 ± 2.74 (10.44 to 12.00)	50	11.94 ± 3.97 (10.81 to 13.07)
Week 4	49	9.43 ± 2.94 (8.59 to 10.27)	50	10.00 ± 3.70 (8.95 to 11.05)
Week 12	46	9.15 ± 3.61 (8.09 to 10.21)	49	8.86 ± 3.28 (7.92 to 9.80)
Week 24	40	8.23 ± 2.67 (7.37 to 9.08)	45	8.40 ± 3.74 (7.28 to 9.52)
EQ-5D-5L VAS				
Baseline	50	66.14 ± 16.85 (61.35 to 70.93)	50	60.14 ± 20.35 (54.36 to 65.92)
Week 4	49	69.94 ± 17.49 (64.92 to 74.96)	50	71.70 ± 15.99 (67.16 to 76.25)
Week 12	46	71.74 ± 17.84 (66.44 to 77.04)	49	74.67 ± 15.38 (70.26 to 79.09)
Week 24	40	74.75 ± 17.17 (69.26 to 80.24)	45	77.62 ± 14.86 (73.16 to 82.09)
EQ-5D-5L Index				
Baseline	50	0.805 ± 0.138 (0.766 to 0.844)	50	0.754 ± 0.197 (0.698 to 0.810)
Week 4	49	0.875 ± 0.117 (0.842 to 0.909)	50	0.862 ± 0.145 (0.821 to 0.903)
Week 12	46	0.872 ± 0.137 (0.832 to 0.913)	49	0.902 ± 0.092 (0.876 to 0.928)
Week 24	40	0.913 ± 0.091 (0.884 to 0.942)	45	0.895 ± 0.159 (0.847 to 0.943)

N, total number of cases; M, mean; CI, confidence interval; SD, standard deviation.

**Table 3:** Effectiveness for all outcomes within treatment groups. Change between baseline and weeks 4, 12, and 24.

	Epidural Administration				Perineural Administration			
Difference to Baseline at	N	M ± SD (95% CI of M)	Cohen’s d	*p*-Value *	N	M ± SD (95% CI of M)	Cohen’s d	*p*-Value *
Numeric Rating Scale (NRS)								
Week 4	49	−2.74 ± 2.24 (−3.38 to −2.09)	−1.22	<0.0001	50	−2.42 ± 2.04 (−3.00 to −1.84)	−1.19	<0.0001
Week 12	46	−2.52 ± 2.51 (−3.27 to −1.78)	−1.00	<0.0001	49	−3.06 ± 2.49 (−3.77 to −2.35)	−1.23	<0.0001
Week 24	40	−3.08 ± 2.12 (−3.75 to −2.40)	−1.45	<0.0001	46	−3.09 ± 2.49 (−3.83 to −2.35)	−1.24	<0.0001
Oswestry Disability Index (ODI)								
Week 4	49	−5.82 ± 6.34 (−7.64 to −3.99)	−0.92	<0.0001	50	−5.82 ± 8.21 (−8.15 to −3.49)	−0.71	<0.0001
Week 12	46	−6.26 ± 7.16 (−8.39 to −4.14)	−0.87	<0.0001	49	−8.96 ± 8.25 (−11.33 to −6.59)	−1.09	<0.0001
Week 24	40	−7.80 ± 8.24 (−10.44 to −5.16)	−0.95	<0.0001	46	−8.78 ± 9.63 (−11.64 to −5.92)	−0.91	<0.0001
Roland Morris Questionnaire (RMQ)								
Week 4	49	−2.53 ± 4.52 (−3.83 to −1.23)	−0.56	0.0003	50	−3.34 ± 4.85 (−4.72 to −1.96)	−0.69	<0.0001
Week 12	46	−2.94 ± 3.95 (−4.11 to −1.76)	−0.74	<0.0001	49	−4.76 ± 5.03 (−6.20 to −3.31)	−0.95	<0.0001
Week 24	40	−3.55 ± 4.49 (−4.99 to −2.11)	−0.79	<0.0001	46	−3.94 ± 5.35 (−5.52 to −2.35)	−0.74	<0.0001
EQ-5D-5L mobility								
Week 4	49	−0.22 ± 1.01 (−0.51 to 0.06)	−0.22	0.1247	50	−0.38 ± 0.85 (−0.62 to −0.14)	−0.44	0.0028
Week 12	46	−0.22 ± 0.84 (−0.47 to 0.03)	−0.26	0.0864	49	−0.59 ± 0.93 (−0.86 to −0.32)	−0.63	0.0001
Week 24	40	−0.38 ± 0.84 (−0.64 to −0.11)	−0.45	0.0073	45	−0.60 ± 1.03 (−0.91 to −0.29)	−0.58	0.0003
EQ-5D-5L self-care								
Week 4	49	−0.14 ± 0.74 (−0.35 to 0.07)	−0.19	0.1806	50	−0.20 ± 0.76 (−0.42 to 0.01)	−0.26	0.0673
Week 12	46	−0.24 ± 0.77 (−0.47 to −0.01)	−0.31	0.0397	49	−0.22 ± 0.94 (−0.50 to 0.05)	−0.24	0.1015
Week 24	40	−0.30 ± 0.79 (−0.55 to −0.05)	−0.38	0.0213	45	−0.29 ± 0.84 (−0.54 to −0.04)	−0.34	0.0263
EQ-5D-5L usual activities								
Week 4	49	−0.41 ± 0.96 (−0.68 to −0.13)	−0.43	0.0044	50	−0.46 ± 0.81 (−0.69 to −0.23)	−0.57	0.0002
Week 12	46	−0.52 ± 0.94 (−0.80 to −0.24)	−0.56	0.0005	49	−0.59 ± 0.98 (−0.87 to −0.31)	−0.61	0.0001
Week 24	40	−0.83 ± 0.81 (−1.09 to −0.57)	−1.01	<0.0001	45	−0.80 ± 0.94 (−1.08 to −0.52)	−0.85	<0.0001
EQ-5D-5L pain/discomfort								
Week 4	49	−0.59 ± 0.93 (−0.86 to −0.32)	−0.63	0.0001	50	−0.56 ± 0.95 (−0.83 to −0.29)	−0.59	0.0001
Week 12	46	−0.63 ± 0.77 (−0.86 to −0.40)	−0.82	<0.0001	49	−0.90 ± 0.96 (−1.17 to −0.62)	−0.93	<0.0001
Week 24	40	−0.88 ± 0.91 (−1.17 to −0.58)	−0.96	<0.0001	45	−0.84 ± 1.09 (−1.17 to −0.52)	−0.78	<0.0001
EQ-5D-5L anxiety/depression								
Week 4	49	−0.33 ± 0.99 (−0.61 to −0.04)	−0.33	0.0249	50	−0.34 ± 1.00 (−0.63 to −0.06)	−0.34	0.0203
Week 12	46	−0.33 ± 1.01 (−0.63 to −0.03)	−0.32	0.0341	49	−0.59 ± 0.96 (−0.87 to −0.32)	−0.62	0.0001
Week 24	40	−0.43 ± 1.01 (−0.75 to −0.10)	−0.42	0.0112	45	−0.56 ± 1.20 (−0.92 to −0.20)	−0.46	0.0033
EQ-5D-5L-based Level Sum Score (LSS)								
Week 4	49	−1.69 ± 3.27 (−2.63 to −0.75)	−0.52	0.0007	50	−1.94 ± 3.18 (−2.85 to −1.04)	−0.61	0.0001
Week 12	46	−2.04 ± 3.14 (−2.97 to −1.12)	−0.65	0.0001	49	−2.90 ± 3.56 (−3.92 to −1.88)	−0.81	<0.0001
Week 24	40	−2.80 ± 3.01 (−3.76 to −1.84)	−0.93	<0.0001	45	−3.09 ± 3.81 (−4.23 to −1.94)	−0.81	<0.0001
EQ-5D-5L VAS								
Week 4	49	3.57 ± 18.69 (−1.80 to 8.94)	0.19	0.1873	50	11.56 ± 18.78 (6.22 to 16.90)	0.62	0.0001
Week 12	46	5.94 ± 17.47 (0.75 to 11.12)	0.34	0.0259	49	14.22 ± 20.49 (8.34 to 20.11)	0.69	<0.0001
Week 24	40	6.70 ± 20.70 (0.08 to 13.32)	0.32	0.0474	45	15.02 ± 19.95 (9.03 to 21.02)	0.75	<0.0001
EQ-5D-5L Index								
Week 4	49	0.0653 ± 0.150 (0.022 to 0.108)	0.44	0.0037	50	0.108 ± 0.167 (0.060 to 0.155)	0.65	<0.0001
Week 12	46	0.0688 ± 0.144 (0.026 to 0.112)	0.48	0.0023	49	0.136 ± 0.163 (0.089 to 0.183)	0.83	<0.0001
Week 24	40	0.0969 ± 0.138 (0.053 to 0.141)	0.70	0.0001	45	0.119 ± 0.191 (0.062 to 0.177)	0.62	0.0001

* Probability value of paired *t*-test. N, total number of cases; M, mean; CI, confidence interval; SD, standard deviation; Cohen’s d, Cohen’s measure of sample effect size within groups.

**Table 4:** Efficacy for all outcomes between treatment groups at weeks 4, 12, and 24.

		Difference between Groups			
Difference at	Ne	Np	M ± SD (95% CI of M)	Cohen’s d	*p*-Value *
Numeric Rating Scale (NRS)					
Week 4	49	50	0.31 ± 2.14 (−0.54 to 1.17)	−0.15	0.4670
Week 12	46	49	−0.54 ± 2.50 (−1.55 to 0.47)	0.22	0.2942
Week 24	40	46	−0.01 ± 2.33 (−1.01 to 0.99)	0.01	0.9811
Oswestry Disability Index (ODI)					
Week 4	49	50	0.00 ± 7.34 (−2.93 to 2.93)	0.00	0.9980
Week 12	46	49	−2.70 ± 7.74 (−5.85 to 0.46)	0.35	0.0929
Week 24	40	46	−0.98 ± 9.01 (−4.86 to 2.89)	0.11	0.6155
Roland Morris Questionnaire (RMQ)					
Week 4	49	50	−0.81 ± 4.69 (−2.68 to 1.06)	0.17	0.3927
Week 12	46	49	−1.82 ± 4.54 (−3.67 to 0.03)	0.40	0.0538
Week 24	40	46	−0.38 ± 4.97 (−2.52 to 1.75)	0.08	0.7211
EQ-5D-5L mobility					
Week 4	49	50	−0.16 ± 0.93 (−0.53 to 0.22)	0.17	0.4087
Week 12	46	49	−0.37 ± 0.89 (−0.74 to −0.01)	0.42	0.0432
Week 24	40	45	−0.23 ± 0.95 (−0.63 to 0.18)	0.24	0.2766
EQ-5D-5L self-care					
Week 4	49	50	−0.06 ± 0.75 (−0.35 to 0.24)	0.08	0.7040
Week 12	46	49	0.01 ± 0.86 (−0.34 to 0.37)	−0.02	0.9341
Week 24	40	45	0.01 ± 0.82 (−0.34 to 0.37)	−0.01	0.9504
EQ-5D-5L usual activities					
Week 4	49	50	−0.05 ± 0.89 (−0.41 to 0.30)	0.06	0.7718
Week 12	46	49	−0.07 ± 0.96 (−0.46 to 0.32)	0.07	0.7223
Week 24	40	45	0.03 ± 0.88 (−0.36 to 0.41)	−0.03	0.8969
EQ-5D-5L pain/discomfort					
Week 4	49	50	0.03 ± 0.94 (−0.34 to 0.41)	−0.03	0.8669
Week 12	46	49	−0.27 ± 0.88 (−0.62 to 0.09)	0.31	0.1398
Week 24	40	45	0.03 ± 1.01 (−0.41 to 0.47)	−0.03	0.8894
EQ-5D-5L anxiety/depression					
Week 4	49	50	−0.01 ± 0.99 (−0.41 to 0.38)	0.01	0.9464
Week 12	46	49	−0.27 ± 0.98 (−0.67 to 0.14)	0.27	0.1913
Week 24	40	45	−0.13 ± 1.11 (−0.61 to 0.35)	0.12	0.5909
EQ-5D-5L-based Level Sum Score (LSS)					
Week 4	49	50	−0.25 ± 3.23 (−1.53 to 1.04)	0.08	0.7054
Week 12	46	49	−0.86 ± 3.36 (−2.22 to 0.51)	0.25	0.2158
Week 24	40	45	−0.29 ± 3.46 (−1.78 to 1.21)	0.08	0.7019
EQ-5D-5L VAS					
Week 4	49	50	7.99 ± 18.73 (0.51 to 15.46)	−0.43	0.0364
Week 12	46	49	8.29 ± 19.09 (0.51 to 16.07)	−0.44	0.0370
Week 24	40	45	8.32 ± 20.30 (−0.45 to 17.10)	−0.41	0.0628
EQ-5D-5L Index					
Week 4	49	50	0.042 ± 0.159 (−0.021 to 0.106)	−0.27	0.1857
Week 12	46	49	0.067 ± 0.154 (0.005 to 0.130)	−0.44	0.0359
Week 24	40	45	0.022 ± 0.168 (−0.051 to 0.095)	−0.13	0.5452

* Probability value of unpaired t-test. Ne, total number of cases (epidural); Np, total number of cases (perineural); M, mean; CI: confidence interval; SD, standard deviation; Cohen’s d, Cohen’s measure of sample effect size between groups.

**Table 5:** Effectiveness for the 3 primary outcomes within treatment groups between baseline and week 24, stratified by type of hernia.

	Epidural			Perineural		
Type of Hernia	N	M ± SD (95% CI of M)	Cohen’s d	N	M ± SD (95% CI of M)	Cohen’s d
Difference from baseline to week 24: Oswestry Disability Index (ODI)						
Bulging	13	−7.31 ± 6.80 (−11.42 to −3.2)	−1.07	7	−8.43 ± 9.11 (−16.85 to −0.01)	−0.93
Extrusion	6	−5.83 ± 5.71 (−11.82 to 0.16)	−1.02	5	−20 ± 10.75 (−33.34 to −6.66)	−1.86
Prolapse	11	−8.82 ± 10.09 (−15.6 to −2.04)	−0.87	25	−5.72 ± 9.08 (−9.47 to −1.97)	−0.63
Residual	8	−7.63 ± 10.31 (−16.24 to 0.99)	−0.74	5	−6.8 ± 2.77 (−10.25 to −3.36)	−2.45
Difference from baseline to week 24: Roland Morris Questionnaire (RMQ)						
Bulging	13	−3.23 ± 4.0652 (−5.69 to −0.77)	−0.79	7	−2.43 ± 3.1 (−5.3 to 0.44)	−0.78
Extrusion	6	−2.83 ± 2.86 (−5.83 to 0.17)	−0.99	5	−10.6 ± 7.02 (−19.32 to −1.88)	−1.51
Prolapse	11	−3.73 ± 4.15 (−6.52 to −0.94)	−0.90	25	−2.88 ± 4.94 (−4.92 to −0.84)	−0.58
Residual	8	−3.63 ± 5.88 (−8.54 to 1.29)	−0.62	5	−3.4 ± 4.39 (−8.86 to 2.06)	−0.77
Difference from baseline to week 24: EQ-5D-5L Index						
Bulging	13	0.07 ± 0.079 (0.022 to 0.118)	0.88	7	0.147 ± 0.119 (0.022 to 0.271)	1.24
Extrusion	6	0.101 ± 0.128 (−0.034 to 0.235)	0.79	5	0.248 ± 0.238 (−0.048 to 0.543)	1.04
Prolapse	11	0.103 ± 0.181 (−0.019 to 0.225)	0.57	25	0.08 ± 0.214 (−0.008 to 0.169)	0.37
Residual	8	0.082 ± 0.15 (−0.044 to 0.207)	0.55	5	0.119 ± 0.105 (−0.011 to 0.249)	1.14

N, total number of cases; M, mean; CI, confidence interval; SD, standard deviation; Cohen’s d, Cohen’s measure of sample effect size within groups.

**Table 6:** Reported adverse events.

Event	Epidural Administration	Perineural Administration
Serious adverse reactions	Ø	Ø
Adverse reactions		
Benign headache	2	Ø
Dizziness	Ø	1

Ø, none.

## Data Availability

The data presented in this study are available on request from the corresponding author.
